# Quantifying Physiological Biomarkers of a Microwave Brain Stimulation Device

**DOI:** 10.3390/s21051896

**Published:** 2021-03-08

**Authors:** Iqram Hussain, Seo Young, Chang Ho Kim, Ho Chee Meng Benjamin, Se Jin Park

**Affiliations:** 1 Center for Medical Convergence Metrology, Korea Research Institute of Standards and Science, Daejeon 34113, Korea; iqram@ust.ac.kr (I.H.); young2da@kriss.re.kr (S.Y.); 2 Department of Medical Physics, University of Science & Technology, Daejeon 34113, Korea; 3 UNITECH CO., Ltd., Seoul 08289, Korea; unitech@unigroup.co.kr; 4 AI Research Group, Sewon Intelligence, Ltd., Seoul 04512, Korea; Hocmb@sewon3h.com

**Keywords:** microwave brain stimulation, physiological biomarker, cognitive workload, wearable bioelectronic medicine

## Abstract

Physiological signals are immediate and sensitive to neural and cardiovascular change resulting from brain stimulation, and are considered as a quantifying tool with which to evaluate the association between brain stimulation and cognitive performance. Brain stimulation outside a highly equipped, clinical setting requires the use of a low-cost, ambulatory miniature system. The purpose of this double-blind, randomized, sham-controlled study is to quantify the physiological biomarkers of the neural and cardiovascular systems induced by a microwave brain stimulation (MBS) device. We investigated the effect of an active MBS and a sham device on the cardiovascular and neurological responses of ten volunteers (mean age 26.33 years, 70% male). Electroencephalography (EEG) and electrocardiography (ECG) were recorded in the initial resting-state, intermediate state, and the final state at half-hour intervals using a portable sensing device. During the experiment, the participants were engaged in a cognitive workload. In the active MBS group, the power of high-alpha, high-beta, and low-beta bands in the EEG increased, and the power of low-alpha and theta waves decreased, relative to the sham group. RR Interval and QRS interval showed a significant association with MBS stimulation. Heart rate variability features showed no significant difference between the two groups. A wearable MBS modality may be feasible for use in biomedical research; the MBS can modulate the neurological and cardiovascular responses to cognitive workload.

## 1. Introduction

Neuroscience deals with understanding the dynamics of cognition, emotion, and behavior in the brain. Researchers are finding ways to assess the brain directly by triggering changes in neural processes that currently affect behavior and basic mental activity [1,2]. Such interventions are usually performed with invasive methods in animal models, which yield detailed presentations of brain-function with high spatial resolution [3]. Unfortunately, the application of most of these methods in healthy humans is very limited. In most human studies, noninvasive brain stimulation (NIBS) techniques are employed to evaluate brain–behavior relationships [4]. Changes in the structure of brain electrical oscillations are responsible for several neurological and mental disorders [5]. The alteration of brain electrical oscillations in a controlled way is expected to be effective for the treatment of such disorders. Neurofeedback also is being utilized as a method for the self-regulation of brain oscillations [6,7].

Transcranial direct current stimulation (tDCS) [8], transcranial magnetic stimulation (TMS) [9], transcranial electrical stimulation (tES) [10], Pulsed Signal Therapy (PST) [11], and microwave brain stimulation (MBS) [12,13,14,15] are the most studied brain stimulation methods that can change or improve the neurological and functional outcome of the brain through noninvasive stimuli. Brain stimulation using contact electrodes is inconvenient for the majority of patients. Noninvasive brain stimulations, such as TMS and tES, produce electric fields that affect relatively wide areas of brain cells compared to invasive methods, such as tDCS. Noninvasive techniques have been studied and implemented to improve and treat neurological disorders.

MBS is a noninvasive method consisting of an antenna or array of antennae focusing an electromagnetic field on a point in the antenna near-field (NF) region [16]. MBS is in the early stages of research, and demands further research [17,18]. The microwave signal can focus the entire cortex thanks to its short-wavelength characteristics, in contrast to other DC currents or low-frequency magnetic fields. The ability to stimulate a large area of the cortex makes the microwave stimulation system an excellent candidate for improving brain functionality and repairing neurological disorders. Most of the electrical stimulation techniques utilize implanted and surface electrodes for brain electrical oscillations. The proposed MBS system is convenient because it does not require the use of implants or surface electrodes attached to the skin. Noninvasive stimulation methods, such as TMS, have the disadvantages of being expensive and creating noise; in contrast, microwave stimulation is cheap and noiseless.

Brain stimulation methods have been successfully adapted for therapeutic outcomes in neurological and psychiatric disorders [1]. Brain stimulation techniques are used as treatments for patients with motor disorders, such as Parkinson disease [19], dystonia [20], and tremor [21]. Brain stimulation is also administered in the treatment of pain and epilepsy [22]. The use of the technique to treat several neurological and psychiatric disorders, such as major depression [23], bipolar disorder, Alzheimer disease [24], and so on, has evolved considerably in recent years.

Wearable bioelectronic devices are now utilized for prognostics [25,26,27], diagnostics of disease, the assistance of treatment of neurological and cardiovascular diseases, stress management [28], and sleep improvement [29]. As the use of NIBS technologies will likely become widespread due to the effectiveness of neuromodulation and the absence of significant adverse events outside of clinical and academic settings, noninvasive brain stimulation systems are now being utilized as wellness and lifestyle devices [30,31].

Brain stimulation has been shown to impact cognitive functions, for example, working memory and attention [32]. As electroencephalography (EEG) is the measure of neurological and functional outcome in the brain, EEG it is used as a quantitative indicator of the effect of brain stimulation [12]. Microwave stimulation is also believed to affect the autonomic nervous system (ANS). As heart rate variability (HRV) is a biomarker of the ANS, analysis of ECG-derived HRV is the key to understanding the relationship between ANS and MBS. In most previous studies, EEG was investigated to understand the neuromodulation triggered by brain stimulation, while cardiovascular and autonomic responses to microwave stimulation have not yet been studied.

We hypothesized that responses of the central nervous system and the cardiovascular system triggered by a MBS system would be immediately sensed by the EEG and ECG circuitry. Signal processing-based feature extraction, followed by statistical data analysis, is likely a reliable method to explore the physiological and functional outcome of brain stimulation.

This study was designed as a double-blind, randomized, sham-controlled study in which physiological ECG and EEG data were recorded, comparing two groups of participants, receiving either active or sham MBS stimuli in conjunction with exposure to a cognitive load in the form of a video game. The rest of this paper is organized into four sections. Section 2 describes the methodology, including details of the MBS system, the study protocol, and data analysis processes. The results are presented in Section 3, followed by a discussion in Section 4. Finally, the conclusions are presented in Section 5.

## 2. Materials and Methods

### 2.1. Microwave Brain Stimulation Device

**Figure 1:**
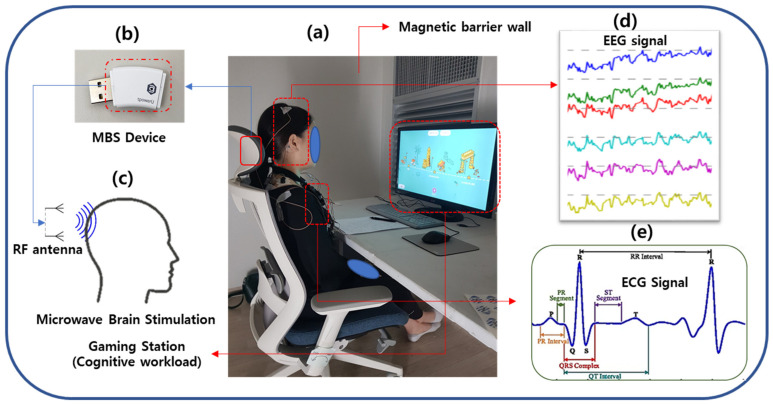
Data acquisition, stimulation, and experimental scenario. (**a**) Experimental scenario and data acquisition system. (**b**) The magnetic brain stimulation device used in this study. (**c**) A simple demonstration of brain stimulation induced by antennas. (**d**) The sample EEG Signals (Fp1, Fp2, C3, C4, O1, O2 positions). (**e**) The sample ECG Signals with fiducial points.

Both active MBS and sham stimuli were administered using a miniature MBS device (tpowerU, Unitech Co., Seoul, Korea) in a noninvasive manner with identical placement and operation, as shown in Figure 1b. This MBS device, held and powered by 5V USB, includes a chip for generating a stimulus wave with a modulator and a radio frequency (RF) probe. The device, positioned in the headrest of a chair, can induce an electrical current in the brain and excite its cell membranes. No additional electrode was placed in the head. It was assumed that the radiated power of modulated microwave stimulation would affect the electrical activity of the neural and cardiovascular systems. As demonstrated in Figure 1c, the active device produces an electromagnetic field using two antennae; one is a 2.4 GHz RF antenna, and the other is a 5.2 GHz RF antenna. The modulation frequency of this device is 35 Hz. The conducted power, i.e., the RF power supplied to the antenna, is 102.57 mW (20.11 dBm) for the 2.4 GHz antenna and 56.75 mW (17.54 dBm) for the 5.2 GHz antenna. The localized specific absorption rate (SAR) for the head is 0.838 W/Kg using the 2.4 GHz antenna and 1.175 W/kg using the 5.2 GHz antenna, both of which are lower than the Institute of Electrical and Electronics Engineers (IEEE) standard [33] and the Korean SAR standard. The brain tissue is exposed to this microwave signal if the subject is positioned in the field of exposure. Sham devices are identical in shape and exterior design but do not generate microwave radiation.

### 2.2. Study Design

**Figure 2:**
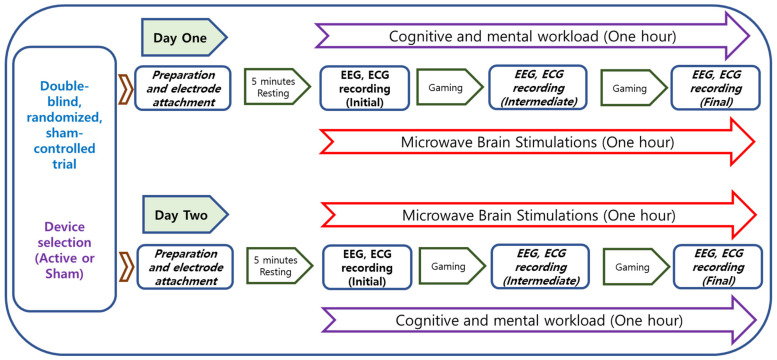
Study design for physiological evaluation of an MBS device as a double-blind, randomized, sham-controlled trial.

The study was conducted according to a protocol approved by the Institutional Review Board of Korea Research Institute of Standards and Science, Daejeon, South Korea. The protocol was designed as a double-blind, randomized, sham-controlled trial. The brief of the experimental protocol is presented in Figure 2. The study was conducted over a total of two days for each participant. The active or sham device is described based on the presence or absence of microwave stimulation in the trial device. The device with active microwave stimulation is described as the Active MBS device, and the device with no stimulation is described as the sham device. Before the start of the experiment, the experimental scenario was explained to the participants. ECG and EEG electrodes were attached to the participants, followed by resting for 10 min, as displayed in Figure 1d,e. After that, participants were required to play a video game for about an hour. The game is a kind of hidden object search, consisting of finding static images and moving images. This video game is designed to demand cognitive and mental effort. During one hour of the cognitive task, the biosignals were measured three times while using active stimulation or the sham device: during the first 5 min (initial phase), from 25 to 30 min (intermediate phase), and 5 min before the end (final phase). A subjective satisfaction evaluation was performed. An example of the experimental scenario is presented in Figure 1a. Room temperature was maintained at 24 °C and relative humidity was 40%.

### 2.3. Randomization

Participants were blindly allocated to the active group or the sham group using a randomization list built in SPSS ver. 24 (IBM, Armonk, NY, USA) with a block randomization method. Devices were numbered in advance by the manufacturer, which did not participate in the investigation. The individuals who distributed the sham and active devices at random did not participate in data acquisition or analysis. Stimulus grouping (active or sham) was revealed for interpretation during the statistical analysis. The participants and clinical staff were blinded to the stimulus type, and each of the participants received one kind of stimulus each day without any knowledge of the device type.

### 2.4. Participants of the Experiment

The participants in this experiment consisted of ten young volunteers (mean age: 26.33 years, 70% male). Participant height distribution was 166.63 ± 6.35 cm, and weight distribution was 65.84 ± 14.49 Kg. The participants had no clinical history of cardiovascular and neurological diseases. ECG and EEG data were recorded at the Center for Medical Convergence Metrology, Korea Research Institute of Standards and Science, Daejeon, South Korea.

### 2.5. Data Acquisition

#### 2.5.1. ECG Data Acquisition

**Figure 3:**
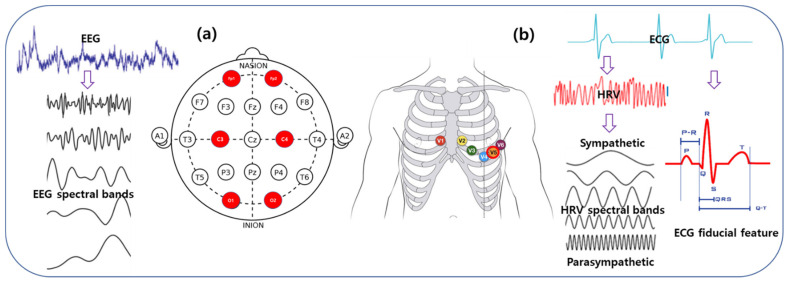
Electrode positions and signal processing of ECG and EEG system. (**a**) Six-Channels EEG electrodes position based on Standard 10-20 EEG system and EEG signal processing methods. (**b**) ECG electrode positioned in V5 lead position and ECG signal processing techniques.

In this study, the ECG was recorded as the representative signal of the cardiovascular system. Single-channel ECG data were continuously acquired using a Biopac MP 160 System (Biopac Systems Inc., Goleta, CA, USA) using the AcqKnowledge ver. 5.0 software. The electrical activity of the cardiac system was recorded using a wireless Biopac BioNomadix RSP and an ECG amplifier (RSPEC-4.3). The amplifier captured the signal using a 3 × 30-cm Electro Lead (BN-EL30-LEAD3) connected to bipolar EL 503 pregelled disposable electrodes attached to the left and right sides of the chests of the participants (Figure 3b). A low-alcohol swab was used to clean the participants’ skin to reduce impedance. In this study, ECG data were collected at the standard V5 lead position. Participants were requested not to consume any drinks such as coffee or alcohol before the tests. During the collection of the ECG data, participants were instructed to stay awake with their eyes open. The temperature of the experiment room was kept at 24 °C, and the relative humidity of that room was maintained at 40%.

#### 2.5.2. EEG Data Acquisition

In this study, the EEG was recorded as the representative signal of the neural system of the brain. Six-channel EEG data were acquired using a Biopac MP 160 Module (Biopac Systems Inc., Goleta, CA, USA) with the AcqKnowledge ver 5.0 software. The electrical activity of the cerebral cortex was continuously recorded using a wireless Biopac BioNomadix EEG amplifier (EEG2-4.3). The amplifier captured the signal via reusable gold-plated 10 mm diameter cup electrodes (EL160) attached to the scalp with a conductive paste (Elefix, Nihon Kohden, Japan). A low-alcohol swab was used to clean the participants’ skin to reduce impedance. In this study, EEG data were taken on the Fp1, Fp2, C3, C4, O1, O2 positions, according to the international 10-20 EEG system, as shown in Figure 3a.

### 2.6. Pre-Processing

All ECG streams were sampled down at 200 Hz to match the optimized sampling rate of the QRS detection algorithms. All premature, missing, or ectopic beats were filtered out. Any 60 Hz AC noise from the local electrical grid was filtered out of the EEG signal. Artifacts of the electrooculography (EOG) and electromyography (EMG) signals were filtered out of the EEG signal.

### 2.7. Feature Extraction

#### 2.7.1. ECG Features

Both the frequency- and time-domain ECG features were derived from the artifact-free heart signal. The frequency-domain analysis, widely described as HRV, is the most studied method for computing the sympathetic and parasympathetic tones of the autonomic nervous system. HRV is derived from the RR interval obtained from R-peaks [34]. The power of the high-frequency band (HF, 0.15–0.4 Hz) of the HRV signal is a measure of parasympathetic activity, while the power of the low-frequency band (LF, 0.04–0.15 Hz) is a biomarker of both sympathetic and parasympathetic tones [34]. LF/(LF+HF) is described as the low-frequency ratio, and HF/(LF+HF) as the high-frequency ratio. Despite the discrepancy [35], the ratio of the two power bands (LF/HF) is described as the degree of sympathetic-vagal balance.

The time-domain analysis provided features based on the fiducial markers of the ECG signal, as shown in Figure 3b. Fiducial points were detected in the ECG waveform using a QRS detector by applying a modified Pan-Tompkins method [36]. As shown in Figure 1e, ECG fiducial features include time interval of the onset, offset, and the peak of P-wave, Q-wave, R-peak, S-wave, and T-wave detected in the ECG signal. RR-I (R-R Interval) is the time gap between the two consecutive R peaks measured in seconds. The QRS complex is the time interval of the onset of the Q-wave and the end of the S-wave in seconds. PRQ is defined as the time interval measured from the start of the P-wave to the end of the Q-wave in seconds. R-height (R-H) is described as the height of the R-wave measured in mV. P-height (P-H) is defined as the height of the P-wave measured in mV. ST interval is defined as the time interval between the end of the S-wave and the start of the T-wave calculated in seconds. QT is described as the time interval measured from the onset of the Q-wave to the closing point of the T-wave measured in seconds. QTc (corrected QT interval) is a QT interval normalized with the RR interval.

#### 2.7.2. EEG Features

EEG may be characterized in terms of frequency and the power within specific frequency bands (Figure 3a), i.e., theta band ranges in 4.0–6.9 Hz, low-alpha wave runs on 6.9–8.9 Hz, high-alpha wave runs on 10.9–12.5 Hz, the low-beta band maintained in 12.9–19.2 Hz, high-beta wave runs on 19.2–32.4 Hz. Various EEG features were extracted from EEG signals using FFT and other techniques to examine the power within the EEG signals. For each time epoch, power spectral density was measured to estimate the power spectrum of that epoch using a Welch periodogram method. From this PSD, the mean power was extracted for each epoch. The mean power was defined as the average power of the power spectrum within the epoch. The epoch width was characterized as 10s.

### 2.8. Statistical Analysis

We compared the demographic data of the participants using descriptive statistics. To evaluate the comparative changes of the physiological features throughout the test, the data were partitioned into intervals indicating the initial resting state, the intermediate state, and the final state. The initial physiological data, recorded at the beginning of the test for five minutes, was considered as the baseline of this study. The relative changes of each phase of stimulation from the initial baseline phase were measured and are presented in a bar chart. Data in the bar chart represent the mean value of each data with a corresponding 95% confidence interval (CI). The independent-samples t-test was used as a comparative measure of the means of data between the active group and the sham group. A *p*-value of less than 0.05 was considered statistically significant. Statistical analyses were performed using SPSS 24 software (IBM, Armonk, NY, USA).

## 3. Results

### 3.1. Effects of MBS on Neurological Outcome

**Figure 4:**
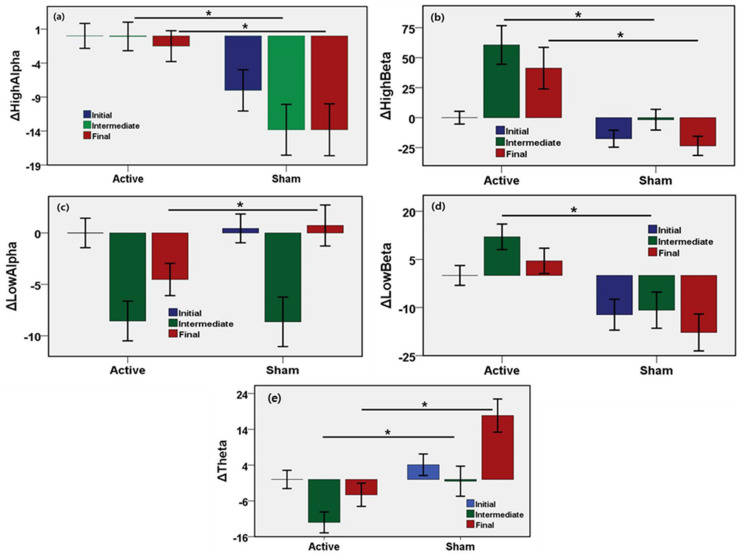
Results from EEG signal analyses for stimulation during the cognitive task. Bar describes the unadjusted mean difference from baseline and error bar as the 96% CI, * indicates *p* < 0.05 (**a**) Increase in power of high-alpha band for active MBS during intermediate and final phase of stimulation (*p* < 0.0001). (**b**) Increase in power of high-beta band for active MBS during intermediate and final phase of stimulation (*p* < 0.0001). (**c**) Decrease in the power of low-alpha band for active MBS during the final phase of stimulation (*p* < 0.0001). (**d**) Increase in power of low-beta band for active MBS during intermediate and final phase of stimulation (*p* < 0.0001). (**e**) Decrease in the power of theta band for active MBS during intermediate and final phase of stimulation (*p* < 0.0001). ∆ means the change relative to the baseline.

To evaluate the changes of neural States induced by active or sham stimulation, brainwave features were measured from the EEG recording in the initial, intermediate, and final phases of the microwave stimulation. The initial neural state was considered as the baseline, and changes of EEG indices of intermediate and the final states with respect to the initial state were evaluated and are shown in Figure 4. The results show the unadjusted mean values, 95% confidence interval, and the *p*-values of the changes of EEG features in the intermediate and the final phase compared to the initial response (Table 1). The mean power of low-alpha wave, high-alpha wave, the low-beta wave, high-beta wave are the most widely used EEG biomarkers of neurological activity. High alpha power increased by 13.7% (95% CI, 9.5–18.0%, *p* = 0.0001) until the middle of stimulation and by 12.3% (95% CI, 7.9–16.7%, *p* = 0.0001) until the end of stimulation after adjustments in the active stimulation relative to the sham group. High beta power increased by 62.3% (95% CI, 44.1–80.5%, *p* = 0.0001) until the middle of stimulation and by 64.8% (95% CI, 45.7–83.8%, *p* = 0.0001) until the end of stimulation after adjustments in the active stimulation relative to the sham group. Low alpha power increased by 0.1% until the middle of stimulation, and decreased by 5.3% (95% CI, −7.8% to −2.7%, *p* = 0.0001) until the end of stimulation after adjustments in the active stimulation relative to the sham group. Low beta power increased by 22.9% (95% CI, 16.0–29.8%, *p* = 0.0001) until the middle of stimulation and by 22.3% (95% CI, 15.4–29.3%, *p* = 0.0001) until the end of stimulation after adjustments in the active stimulation relative to the sham group. Theta power decreased by 11.5% (95% CI, −16.6% to −6.4%, *p* = 0.0001) until the middle of stimulation, and by 22.1% (95% CI, −27.8% to −16.5%, *p* = 0.0001) until the end of stimulation after adjustments in the active stimulation relative to the sham group. There were significant differences in the power of high alpha, high beta, low beta, theta during the cognitive task between the active and the sham group. No statistical significance was found in low alpha power only during the middle of stimulation.

### 3.2. Effects of MBS on Autonomic Nervous System

**Figure 5:**
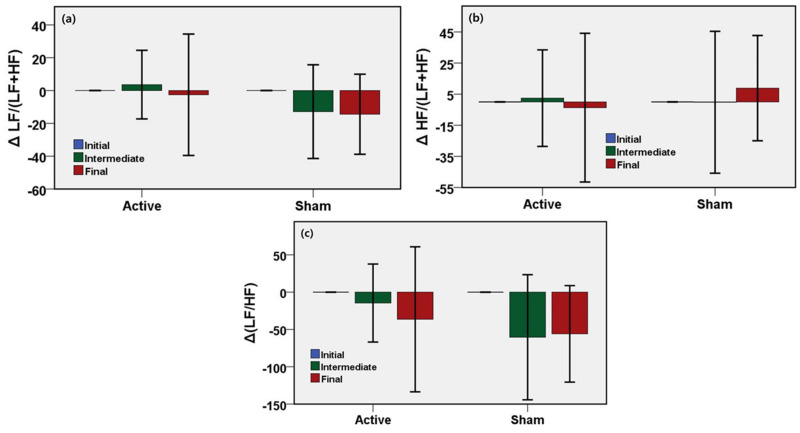
Results from HRV analyses for stimulation during the cognitive task. Bar describes the unadjusted mean difference from baseline and error bar as the 96% CI. (**a**) No significant difference was found in changes of LF ratio between active and sham devices. (**b**) No significant difference was found in changes of HF ratio between active and sham devices. (**c**) No significant difference was found in changes of LF/HF between active and sham devices. ∆ means the change relative to the baseline.

To investigate the physiological changes induced only by active or sham stimulation, the protocol included the administration of stimulation in the presence of cognitive load (computer game). To understand the effect of stimulation on autonomic tone during a cognitive task, ECG data were recorded during the start of the task, the middle of the task, and the end of the task. ECG-derived HRV is the indicator of the state of the autonomic nervous system. The initial autonomic state was considered as the baseline and changes of HRV indices of intermediate and the final states with respect to the initial state were evaluated and are shown in Figure 5. Table 2 shows the unadjusted mean values, 95% confidence interval, and the *p*-values of the changes of HRV response in the intermediate and the final phase compared to the initial response. LF/(LF+HF) is an HRV index and biomarker of the autonomic function that reflects the sympathetic activity of ANS. LF ratio increased by 16.5% until the middle of stimulation, and by 11.9% until the end of stimulation after adjustments in the active stimulation relative to the sham group. On the other hand, HF/(LF+HF) reflects the parasympathetic or vagal activity. The HF ratio increased by 2.6% until the middle of stimulation and decreased by 12.4% until the end of stimulation in the active group compared to the sham group. LF/HF is an HRV parameter indicating the sympathetic-vagal-balance in the ANS. The HF ratio increased by 45.9% until the middle of stimulation and increased by 19.5% until the end of stimulation in the active group compared to the sham group. No significant difference was found in the HRV measures during the cognitive task between the active and the sham group.

### 3.3. Effects of MBS on ECG Fiducial Features

**Figure 6:**
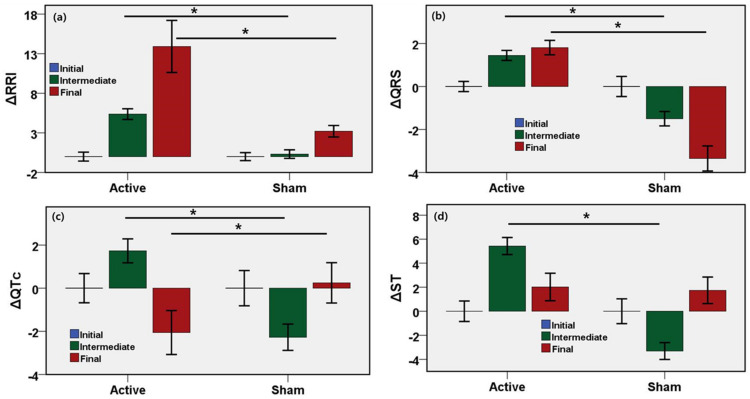
Results from ECG signal analyses for stimulation during the cognitive task. Bars describe the unadjusted mean difference from baseline and error bar as the 96% CI, * indicates *p* < 0.05. (**a**) Increase in RR Interval for active MBS during intermediate and final phase of stimulation (*p* < 0.0001). (**b**) Increase in QRS interval for active MBS during intermediate and final phase of stimulation (*p* < 0.0001). (**c**) Increase in QTc for active MBS during intermediate (*p* < 0.0001) and decrease in QTc for active MBS during the final phase of stimulation (*p* < 0.001). (**d**) Increase in ST for active MBS during the intermediate phase of stimulation (*p* < 0.001). ∆ means the change relative to the baseline.

To investigate the changes of ECG fiducial features induced by active or sham stimulation, ECG time-domain interval features were measured from the ECG recording in the initial, intermediate, and final phases of the microwave stimulation. The initial cardiac state was considered as the baseline, and changes of ECG indices of intermediate and the final states with respect to the initial state were evaluated and are shown in Figure 6. The results show the unadjusted mean values, 95% confidence interval, and the *p*-values of the changes of ECG fiducial features in the intermediate and the final phase compared to the initial response (Table 3). RR interval, QRS interval, ST, QT, QTc are the most common ECG fiducial features and biomarkers of cardiovascular activity. The RR interval increased by 5.1% (95% CI, 4.2–5.9%, *p* = 0.0001) until the middle of stimulation and by 10.7% (95% CI, 7.3–14.1%, *p* = 0.0001) until the end of stimulation after adjustments in the active stimulation relative to the sham group. The QRS interval increased by 2.9% (95% CI, 2.5–3.3%, *p* = 0.0001) until the middle of stimulation and by 5.2% (95% CI, 4.5–5.8%, *p* = 0.0001) until the end of stimulation after adjustments in the active stimulation relative to the sham group. QTc interval increased by 4.0% (95% CI, 3.2–4.8%, *p* = 0.0001) until the middle of stimulation and decreased by 2.3% (95% CI, −3.7% to −0.9%, *p* = 0.0001) until the end of stimulation after adjustments in the active stimulation relative to the sham group. ST interval of the active group increased by 8.7% (95% CI, 3.2–4.8%, *p* = 0.0001) until the middle of stimulation relative to the sham group. RRI, QRS, and QTc were statistically significant and associated with the changes due to microwave stimulation. Although QT and ST interval significantly changed in the active group relative to the sham group until the intermediate phase, no significant changes were found in the QT or ST until the end of stimulation during the cognitive task between the active and the sham group.

## 4. Discussion

In our study, we aimed to characterize the neural, cardiac, and autonomic changes of humans due to microwave brain stimulation. The extent of change was shown to be dependent on the type of stimulation and the strength of SAR value. As a wellness device outside of a clinical setting, the SAR value needs to remain within a safe range in order to conform to the regulations of each country. We evaluated the neurological and cardiovascular variations resulting from brain stimulation during a cognitive task. As the sham device possessed no stimulation, the sham condition was characterized as a nonstimulus case, and represented the nonstimulus cognitive stressor. On the other hand, the active device inducing stimulation during the cognitive task represented the stimuli-induced cognitive stressor.

Cognitive performance demands attention and concentration. The high-alpha power is affected by cognitive workload. Small power in the high-alpha band is an indicator of the high cognitive load which is required for good performance [37,38]. In this study, the sham group showed a high-alpha power decrease during the intermediate and final phases of the cognitive task. The high-alpha power attenuates over time due to memory and mental workload, and induces stress. Additionally, high-alpha is an indicator of a lack of stress and relaxation [39]. The high-alpha power needs to remain elevated to induce relaxation during the cognitive and mental tasks. In this study, a big reduction of the high-alpha power was observed for the sham device, while power in the high-alpha band remained stable for the active stimulation. This provides evidence of a positive neuromodulation induced by the MBS during the cognitive task.

Power in the low-alpha band and theta-band showed negative correlations to cognitive performance. Large power in the low-alpha band and the theta band is a marker of low attention. Furthermore, theta power is related to drowsiness, inversely affecting the performance of the task [40]. In this study, the sham group displayed an increase in low-alpha and theta power during the final phase of the cognitive task. On the other hand, the active group showed a drop in the final phase of the task. Both of the groups displayed a drop in the low-alpha power during the intermediate phase. A cognitive workload negatively affects the performance over time and tends to drop the power in high-alpha frequency. In the case of the active stimulation, power in the high-alpha band remained stable during the intermediate phase and dropped slightly at the end of the task compared with the initial phase.

Beta power is considered a positive indicator of attention [41]. Low-beta power is a marker of alertness of the neural system. In this study, low beta power decreased continuously during the middle and the final periods of the cognitive event. In contrast, the active group demonstrated a rise in low-beta power throughout the task. High-beta power tends to indicate a high degree of alertness, which may be related to stress. The higher amplitude of high-beta power can induce stress in the neural state. The active group showed an increase in high-beta in the intermediate period of the experiment, followed by a small drop later.

In this study, no difference was found between the active MBS and the sham group in the ECG-derived HRV measures for a small interval during the middle and end periods of microwave stimulation. HRV is considered as a measure of the autonomic activity of the body. Frequency-domain HRV features are easy to measure from a short interval of ECG data. Among HRV features, LF describes sympathetic and parasympathetic activity. LF reflects the vasomotor activity and increases with activity and stress. On the other hand, the HF indicates respiratory activity and increases with relaxation [31,42]. There was no quantitative evidence of change of HRV due to active MBS.

The current study found RRI and QRS to be measures of the effect of active MBS on cardiovascular activity, and showed significant differences between the active MBS group and the sham group. The RR interval of the active MBS group increased in the intermediate and final phases of the microwave stimulation compared with the sham group. The ECG QRS interval also increased in the active MBS group compared with the sham group. Other ECG fiducial measures did not show any difference between active MBS and the sham group.

As shown in Table 4, the studied brain stimulation technique was shown to be advantageous due to its noninvasive characteristics compared with the other microwave stimulation methods. As microwaves have a short skin depth, they can be concentrated at a specific area of the brain [14]. Microwave stimulation has been shown to modulate neural activity [15,17] and increase nerve cell firing rate [14,18]. The EEG spectral bands showed no significant change for microwave brain stimulation [12]. However, the effects of microwave stimulation on cardiovascular and autonomic function, as studied here, had not been explored in other studies. Other methods have administered stimulation as doses over a certain period. However, the present approach can be applied continuously over a more extended period.

Although we analyzed global EEG data based on the frontal lobe, occipital lobe, and parietal lobe together in order to better understand changes in EEG due to microwave stimulation, we did not study each lobe separately to understand the effect of MBS stimulation in the presence of a cognitive stressor. Although the entire cortex strongly resembles individual lobes, there are still specific cognitive and functional outcomes on each cortical lobe. For this reason, this study generalized to only the entire cortex. Participants were instructed to perform a cognitive task, i.e., to play a video game, in a steady manner. Any attempt to accelerate the task would rise to variations in the neurological measurements. As the MBS device was positioned in the headrest of the chair close to the occipital lobe, any movement during the test had the potential to shift the microwave field of the stimulator. This simulation technique was investigated with a small group of participants, and further research in a clinical setting is required. The physiological effects may vary with the intensity of the magnetic field induced by the microwave; this will be investigated in the future.

## 5. Conclusions

The neurological and cardiovascular effects of microwave stimulation devices can be quantified through the use of wearable physiological sensing devices. The neural features, power of high-alpha, high-beta, low-beta bands, low-alpha and theta wave, and cardiac features, RR interval, QRS interval, were shown to be significant biomarkers which could be modulated with noninvasive microwave stimulation. The MBS device was shown to enhance neurological and cardiovascular responses to cognitive workload. This stimulation method has broad potential to improve cognitive performance, reduce stress, and excite brain rhythms in long-term monitoring outside of a clinical setting. This noninvasive stimulation technique is a promising candidate for wearable bioelectronics medicine and further neuroscience research.

## 6. Patents

This manuscript is related to the Korean patent titled “System For Reducing Specific Absorption Rate”; patent number KR1020040045160.

## Figures and Tables

**Table 1:** Results of the statistical analysis of the EEG features of the Active MBS and the Sham group for stimulation during the cognitive task. The initial phase is considered as the baseline. * indicates *p* < 0.05.

EEG Feature	Phase	Mean Value		Change from Baseline (%)			
		Active	Sham	Active	Sham	Difference	*p*-Value
High Alpha (Relative Power)	Baseline	0.10	0.11	-	-	-	-
	Intermediate	0.10	0.11	−0.08	−13.82	13.74	0.0001 *
	Final	0.10	0.11	−1.51	−13.82	12.31	0.0001 *
Low Beta (Relative Power)	Baseline	0.12	0.15	-	-	-	-
	Intermediate	0.13	0.15	12.05	−10.83	22.88	0.0001 *
	Final	0.12	0.14	4.55	−17.79	22.33	0.052
Low Alpha (Relative Power)	Baseline	0.20	0.18	-	-	-	-
	Intermediate	0.18	0.16	−8.56	−8.64	0.08	0.96
	Final	0.19	0.18	−4.53	0.72	−5.25	0.0001*
Theta (Relative Power)	Baseline	0.46	0.38	-	-	-	-
	Intermediate	0.41	0.37	−12.01	−0.50	−11.50	0.0001 *
	Final	0.44	0.42	−4.29	17.84	−22.13	0.0001 *
High Beta (Relative Power)	Baseline	0.12	0.18	-	-	-	-
	Intermediate	0.18	0.21	60.62	−1.68	62.31	0.0001 *
	Final	0.15	0.17	41.28	−23.49	64.76	0.0001 *

**Table 2:** Results of the statistical analysis of the HRV features of the Active MBS and the Sham group for stimulation during the cognitive task. The initial phase is considered as the baseline.

HRV Feature	Phase	Mean Value		Change from Baseline (%)			
		Active	Sham	Active	Sham	Difference	*p*-Value
LF/(LF+HF)	Baseline	0.59	0.63	-	-	-	-
	Intermediate	0.60	0.60	3.62	−12.85	16.47	0.32
	Final	0.59	0.58	−2.56	−14.44	11.87	0.51
HF/(LF+HF)	Baseline	0.41	0.37	-	-	-	-
	Intermediate	0.40	0.40	2.44	−0.21	2.65	0.92
	Final	0.41	0.42	−3.68	8.87	−12.55	0.61
LF/HF	Baseline	2.17	2.66	-	-	-	-
	Intermediate	1.70	1.97	−14.59	−60.44	45.85	0.33
	Final	1.86	1.80	−36.40	−55.92	19.52	0.69

**Table 3:** Results of the statistical analysis of the ECG fiducial features of the Active MBS and the Sham group for stimulation during the cognitive task. The initial phase is considered as the baseline. * indicates *p* < 0.05.

ECG Fiducial Feature	Phase	Mean Value		Change from Baseline (%)			
		Active	Sham	Active	Sham	Difference	*p*-Value
RR Interval, s	Baseline	0.79	0.86	-	-	-	-
	Intermediate	0.83	0.87	5.37	0.32	5.05	0.0001 *
	Final	0.90	0.89	13.91	3.21	10.70	0.0001
QRS Interval, s	Baseline	0.11	0.11	-	-	-	-
	Intermediate	0.11	0.11	1.44	−1.50	2.94	0.0001 *
	Final	0.11	0.11	1.81	−3.35	5.16	0.0001 *
QT Interval, s	Baseline	0.45	0.43	-	-	-	-
	Intermediate	0.47	0.42	4.44	−2.33	6.77	0.0001 *
	Final	0.45	0.44	−0.67	1.16	−1.83	0.08
QTc Interval, s	Baseline	0.51	0.46	-	-	-	-
	Intermediate	0.52	0.45	1.73	−2.27	4.01	0.0001 *
	Final	0.49	0.46	−2.06	0.25	−2.30	0.0001 *
ST Interval, s	Baseline	0.36	0.34	-	-	-	-
	Intermediate	0.38	0.33	5.43	−3.31	8.74	0.0001 *
	Final	0.36	0.35	2.02	1.74	0.28	0.732

**Table 4:** Comparison of microwave brain stimulation methodologies and results between proposed work and previous works.

Study	Stimulation Characteristics	Study Sample	Physiology Domain	Outcomes
Suhhova et al. [17]	450 MHz microwave stimulation with 25 Hz modulation frequency at two exposure rates	15 healthy volunteers	EEG frequency spectral domain	Change in the power of alpha, beta1, and beta2 with exposure level.
Bachmann et al. [15]	450 MHz microwave stimulation with 40 Hz harmonics	14 healthy subjects	EEG frequency spectral domain	Increase in power of 10 Hz, 20 Hz, and 30Hz compared to the sham (without) stimulation.
Seo et al. [14]	6.5 GHz microwave stimulation with a skin-contact stimulator.	One mouse	Action potential and Nerve firing rate (FR)	FR excited during 1 Hz stimulation and inhibited during 50 Hz stimulation.
Beason et al. [18]	900 MHz microwave stimulation using cell phone at 217 Hz modulation.	34 adult birds (zebra finches)	Action potential and Nerve firing rate (FR)	FR increased for 76%of the responding cells.
Hinrikus et al. [12]	400 MHz microwave stimulation with 7 Hz on-off modulation.	20 healthy volunteers	EEG frequency spectral domain	No significant changes were found in EEG bands power.
Proposed work	2.4 GHz and 5.2 GHz microwave stimulation with 35 Hz modulation.	10 healthy volunteers (Double-blind sham-controlled trial)	EEG frequency spectral domain, ECG time domain, and HRV time and frequency-domain analysis	Significant association of EEG spectral band power and ECG fiducial features are found with active microwave simulation relative to sham group. No association found for HRV features.

## Data Availability

Not applicable.
